# Characteristics of the Surface Topography and Tribological Properties of Reinforced Aluminum Matrix Composite

**DOI:** 10.3390/ma15010358

**Published:** 2022-01-04

**Authors:** Magdalena Niemczewska-Wójcik, Manickaraj Pethuraj, Marimuthu Uthayakumar, Mohd Shukry Abdul Majid

**Affiliations:** 1 Faculty of Mechanical Engineering, Cracow University of Technology, Jana Pawla II 37, 31-864 Cracow, Poland; 2 Faculty of Mechanical Engineering, Younus College of Engineering and Technology, Kollam 691010, Kerala, India; mrajcad@gmail.com; 3 Faculty of Mechanical Engineering, Kalasalingam Academy of Research and Education, Krishnankoil 626126, Tamil Nadu, India; uthaykumar@gmail.com; 4 Faculty of Mechanical Engineering Technology, University Malaysia Perlis, Perlis 02600, Malaysia; shukry@unimap.edu.my

**Keywords:** aluminum matrix composite, surface topography, block-on-ring tester, tribological characteristics, friction coefficient, linear wear, wear intensity

## Abstract

Due to their excellent synergistic properties, Aluminum Matrix Composites (AMC) have achieved a high degree of prominence in different industries. In addition to strength, the wear resistance of materials is also an important criterion for numerous applications. The wear resistance depends on the surface topography as well as the working conditions of the interacting parts. Therefore, extensive experiments are being conducted to improve the suitability of engineering materials (including AMC) for different applications. This paper presents research on manufactured aluminum metal matrix composites reinforced with 10 wt.% of Al_2_SiO_5_ (aluminum sillimanite). The manufactured and prepared samples were subjected to surface topography measurements and to tribological studies both with and without lubricant using a block-on-ring tester. Based on the results, analyses of the surface topography (i.e., surface roughness parameters, Abbott–Firestone curve, and surface defects) as well as of the tribological characteristics (i.a. friction coefficient, linear wear, and wear intensity) were performed. Differences in the surface topography of the manufactured elements were shown. The surface topography had a significant impact on tribological characteristics of the sliding joints in the tests where lubrication was and was not used. Better tribological characteristics were obtained for the surfaces characterized by greater roughness (determined on the basis of both the profile and surface texture parameters). In the case of tribological tests with lubrication, the friction coefficient as well as the wear intensity was significantly lower compared to tribological tests without lubrication. However, lower values of the friction coefficient and wear intensity were still recorded for the surfaces that were characterized by greater roughness. The obtained results showed that it is important to analyze the surface topography because surface characteristics influence tribological properties.

## 1. Introduction

Aluminum and its alloys are lightweight materials that have been used extensively in the aerospace and automobile industries due to their superior properties, such as their higher strength to weight ratio, excellent performance at low temperatures, and corrosion resistance [1]. However, they have drawbacks such as poor wear resistance and lower high-temperature performance. To eradicate these problems, aluminum alloys, which are known as Metal Matrix Composites (MMCs), with ceramic particles added as reinforcement have been developed [2]. Some of the most commonly used ceramic particles are Al_2_O_3_, SiC, and B_4_C [3,4,5], all of which increase the wear resistance and have led to the application of these materials in automotive and aircraft components. Manufacturers such as Duralcan, Martin Marietta, and Lanxide have used Al/SiCp composites for fabricating pistons, brake rotors, and propeller shafts, while Toyota and Dupont use Al/Al_2_O_3_ to make their piston rings and connecting rods [6]. 

Even though these composites possess good wear resistance, they are subjected to oxidation or corrosion due to prolonged usage and exposure to the atmosphere. To overcome this issue, naturally available minerals such as Bauxite [7], Rutile [8], Talc [9], and Mica [10] can be used as reinforcements. Sillimanite (Al_2_SiO_5_) is one such mineral that has high hardness, modulus, corrosion resistance, thermal stability, and a low thermal expansion coefficient [11]. These tailormade properties enable them to be used in automotive applications such as brake drums, pistons, and cylinder liners, among others, because of their high wear and thermal resistance properties.

In their paper, [12] Singh et al. developed Al/Al_2_SiO_5_ MMCs using the stir casting method with various reinforcement sizes and performed mechanical and wear tests on them. The composites had a strong bond between the particles, which resulted in increased hardness and wear resistance in contrast with the basic alloy. Two-body wear tests implied that reinforcement of 25 µm was less resistant to wear than plain aluminum, whereas a mesh size of 200 µm displayed an increased wear pattern [13]. Sharma et al. evaluated the effect of particle size on the wear resistance property of a sillimanite-reinforced composite fabricated by stir casting. A wear test was designed and conducted for three different particle sizes. Particles of 1–20 μm had a higher wear strength (55% greater) compared to the monolithic aluminum alloy. Other particles of 32–50 μm and 75–106 μm had no impact on wear rates [14]. Kurt et al. analyzed the influence of alumina particles on the abrasive wear resistance of Al/Al_2_O_3_ composites and demonstrated decreased abrasive wear resistance due to the accumulation of oxides in the Al particles from the samples that had been treated with high-energy ball milling [15].

Baradeswaran et al. [16] witnessed an increase in the wear rate of the Al/Al_2_O_3_ composites when the load was increased. The wearing loss at an elevated load increased for all wt.% of Al_2_O_3_ at a constant speed of 0.6 m/s. This was because an oxide film was no longer observed within the specified load range on the wear surface. The pure alloy demonstrated maximum wear loss, while a minimum wear loss was recorded in 6 wt.% Al_2_O_3_-strengthened composites. In a similar study using B_4_C as a reinforcement material, the author witnessed an increased wear resistance when the reinforcement was at higher wt.%. The reason for controlling the wear rate of the composites is the Mechanical Mixed Layer (MML) that forms on the worn surface [17]. 

A review of the literature shows that there are many works presenting test results that have been obtained for different materials, including coatings and Aluminum Metal Matrix Composites. Most of the works concern the study of material properties, mechanical properties [18,19,20] and microstructure, and their influence on wear [20,21,22,23,24,25] (tribological characteristics—friction coefficient, wear traces). Usually, if a surface analysis is conducted, only profile roughness parameters (such as Ra or Rz) are checked, which is not enough to characterize the surface topography, a fact that the authors Brown et al. [26], Leach [27], Niemczewska-Wójcik et al. [28], and Whitehouse [29] have drawn attention to. However, there are few studies presenting the results of wider surface topography studies and their influence on tribological characteristics. The same is true for studies of tribological properties of composites that have been reinforced with aluminum sillimanite that have been carried out under various conditions (without and with lubrication).

The innovation of this work is the presentation of surface topography studies prior to tribological studies. The aim of the experimental studies was to check how two differently shaped surfaces (including different surface roughness as well as Abbott–Firestone curve parameters and surface defects) of the studied material (AMC) affect the tribological characteristics (friction coefficient and wear intensity) in two tribological study conditions (without and with lubricant). There is no such analysis in the literature. Most researchers only focus their attention on preparing samples [30] according to standards, carrying out tribological tests, and checking the worn surface after tribological tests [31]—this involves assessing the traces of wear on the basis of microscopic images (scanning electron microscope—SEM, optical microscope—OM) and wear products (SEM/EDS—energy dispersive spectroscopy).

Therefore, within the scope of this work, surface topography (surface morphology and surface texture parameters) analysis and tribological characteristic (friction coefficient, linear wear, wear intensity) analysis were conducted. 

The tribological tests and surface topography measurements were repeated many times (at least three times). Due to the number of obtained results and their repeatability, the representative results are shown in tables and figures.

## 2. Materials and Methods

### 2.1. Material Characteristics—Manufacturing Process and Properties

The subjects of the study were samples made of aluminum sillimanite-reinforced composite—ASRC (Aluminum Matrix Composite with 10% reinforcement Al_2_SiO_5_). The characteristics of the studied material are presented in Table 1.

Pure aluminum was used as a matrix material and had a purity of 99.5%. Pure aluminum can be complemented by sillimanite used as a reinforcement material. The average size of the sillimanite particle was in the range of 55 to 80 µm (data provided by VV Minerals, India). The vacuum casting method was used to fabricate the composite for the designed reinforcement level. The required amount of aluminum was heated up to 750 °C and was maintained for one hour in an electrical induction furnace (SwamEquip, Chennai, Tamil Nadu, India). The reinforcement particles were pre-heated to 500 °C to remove the moisture content and to maintain thermal equilibrium during mixing with the molten aluminum. The heated reinforcements were slowly added to the molten metal and were stirred for 5 min. A small amount of magnesium (5 wt.%) was also added to enhance the wettability of the materials. The molten metal was poured in a preheated hot mould (ranging between 150 and 200 °C). The metal mould was attached to a vacuum pump—double stage pump (SwamEquip, Chennai, Tamil Nadu, India) that removed the air, which helped the composites to become unsullied. The size of the manufactured specimens was that of a 300 × 300 × 10 mm^3^ plate.

**Figure 1:**
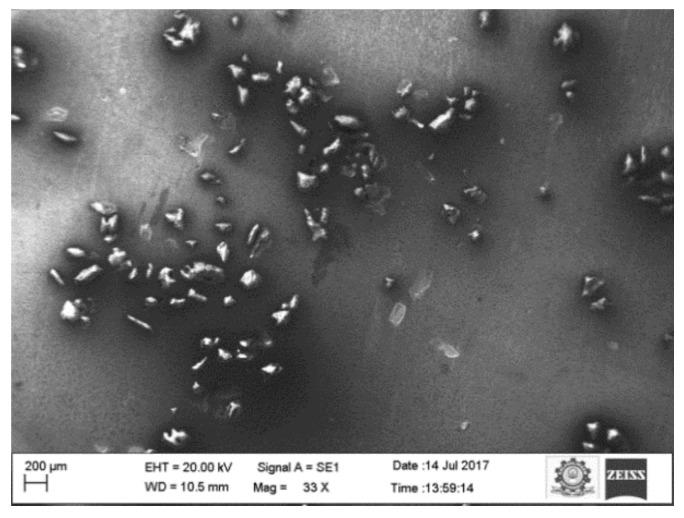
SEM image of 10 wt.% aluminum sillimanite reinforced composites.

To ensure the uniform and homogenous distribution of the reinforcement particles, SEM (Carl Zeiss, One North Broadway, NY, USA) with a suitable accelerating voltage was applied. Back-Scattered Electron (BSE) mode was also used to observe the distribution of the reinforcement particles. Prior to metallographic studies, the specimen was perfectly polished in the polishing machine using SiC paper of various sizes. To observe the microstructure of the aluminum and its composites, the Keller reagent was prepared as per the standards and was used as an etching agent. Figure 1 shows a micrograph of the reinforced composites at 10 wt.%. The SEM images prove the uniform distribution of the reinforcement particles in the aluminum matrix.

The composite materials were cut as per ASTM standard D3532-12 and D256 to perform the tensile and impact tests. The experiments were repeated three times; the average values of the tensile and impact strengths of the composite were reported. The hardness was also measured for all of the composites. A micro-Vickers hardness testing machine (Mitutoyo South Asia Pvt. Ltd., New Delhi, India) with a diamond indenter (136°) was used to measure the microhardness of the composites. Measurements were taken at five locations, and the average hardness value was reported.

The addition of ceramic particles to aluminum alloys leads to improvements in hardness. According to Seah et al. [32] and Sahin [33], the increased hardness can also be attributed to the fact that the hard sillimanite particles act as barriers to the free movement of the dislocations within the matrix. The increase in the tensile and impact strength is due to the presence of sillimanite particles that impart strength to the matrix alloy, thereby providing enhanced resistance to tensile stresses [34]. Ünlü [35] also made similar observations during when testing Al-Al_2_O_3_ composites.

Then, the specimens that were thus manufactured were cut to the required size. After cutting, the specimens were polished in a finishing process. As a result of this treatment, two types of surface characteristics were obtained—A (time of polishing—30 min) and B (time of polishing—15 min).

### 2.2. Research Methodology—Experimental and Measurement Methods

Within the scope of this experimental work, surface topography measurements and tribological studies were carried out. These studies aimed to show how the surface characteristics (texture) of the samples and the cooperation conditions influence the results, namely the tribological characteristics (friction coefficient and wear intensity).

Surface topography studies were carried out using a white light interference microscope WLIM (Talysurf CCI, Taylor Hobson Ltd., Leicester, UK). A Mirau 10× lens was used, thanks to which 1.65 mm × 1.65 mm measured areas were obtained (analysed area—1.0 mm × 1.0 mm). The measured surfaces were prepared for analysis using the advanced metrology software Talymap Platinium v.7.1 (Taylor Hobson Ltd. in cooperation with Digital Surf, Leicester, UK)—a spatial filter was used to remove noise, and thresholding was used to reduce the extremes of height and depth in the surface. 

Because the samples had a cuboid shape and a flat surface, no shape filtering was used. Additionally, waviness was not removed because it plays as an important role in the operation process as roughness and is an integral part of the surface topography (surface texture for flat elements). The parameters describing the Abbott–Firestone curve were not filtered, reflecting the real characteristics of a surface subjected to the operation process. The waviness was automatically filtered (Gaussian filter) when the surface roughness parameters (Sq, Ssk, Sku, Sp, Sv) were generated. Based on the obtained results, a qualitative analysis (photo, surface map, surface axonometric, series of profiles) and quantitative analysis (Sk parameters, surface roughness parameters) were carried out, the results of which are presented in Section 3, Results and Discussion.

Tribological tests were carried out on a block-on-ring wear tester (Łukasiewicz Research Network—The Institute for Sustainable Technologies, Radom, Poland) to evaluate the lubricants and engineering materials [36,37]. The details (tribotester view, sliding joint, and studies parameters) of the tribological tests are presented in Table 2.

The friction pair consisted of a stationary block (Surfaces A and B, where A is the less rough surface and B is the rougher surface) pressed at a load of 20 N against a ring rotating at a speed of 0.1 m/s.

The studies aimed to show the influence of the surface characteristics of the samples made of ASRC material and the tribological test conditions (without lubrication, with lubrication) on tribological characteristics.

## 3. Results and Discussion

The results of the studies are presented in the form of tables and graphs. Table 3 presents the results of surface topography studies for two types of surfaces—those that characterized by lesser and greater roughness, which is described by parameter Ra—the arithmetical mean height of the profile (a parameter commonly used to assess surfaces). The value of the Ra parameter was determined for the 1024 profiles that were generated (using the advanced metrology software; function *convert surface into series of profiles*) and hence the given average value and standard deviation, which allowed us to conclude the measured areas of the tested samples—for Surface A, the Ra parameter was 0.095 µm ± 0.007, and for Surface B, the Ra parameter was 0.218 µm ± 0.086.

Since the Ra parameter is an average parameter (given in standards), it does not show sensitivity to local peaks or pits. Therefore, based on experience from previous work [28,38], an analysis of the 3D parameters describing the surface topography was performed. This allowed for the characterization of the tested surfaces in terms of shape and the potential influence of these on tribological properties.

The analysis of the surface topography of the studied samples showed clear differences in the formation of irregularities. On Surface A, regular scratches and a few valleys were visible, the characteristics of which are given in Table 4. On Surface B, regular scratches were less visible but also occurred on the surface. The characteristic peaks and pits, locally located, became more visible; these characteristics are also presented in Table 4.

Both peaks and pits can significantly affect the results of tribological tests. For friction pairs without lubrication, pits (valleys/cavities) can accumulate wear products. In the case of friction pairs with lubricant, valleys can be both local places of accumulation for the lubricating medium as well as wear products. On the other hand, local peaks can be places where (bearing Surface B was smaller than bearing Surface A) the ASRC sample surface can be in direct contact with the surface of the counter sample 100Cr6. 

**Figure 2:**
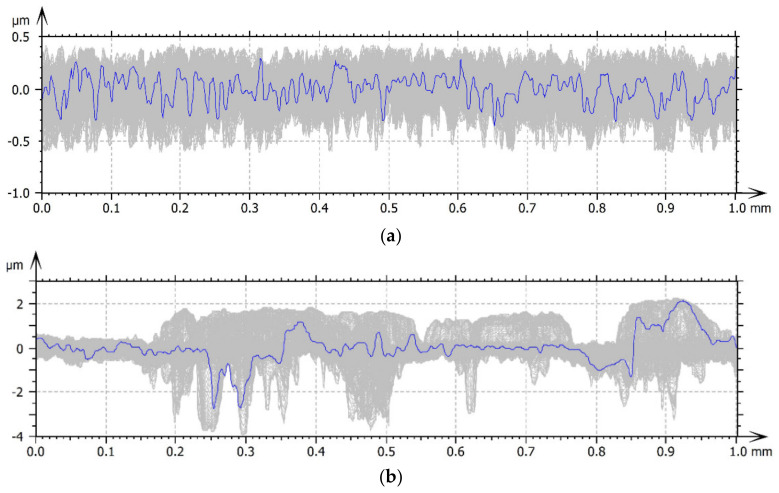
Series of profiles: (**a**) Surface A, (**b**) Surface B.

Figure 2 presents a series of 1024 profiles (marked in grey color) that were generated for the studied surfaces along with the selected profiles (marked in blue color).

Profile analysis showed that Surface A was characterized by unevenness in the form of peaks and pits, with much smaller dimensions than those that were noted for Surface B.

Table 4 shows selected fragments of the studied surfaces. Characteristic peaks and pits that could have played a significant role in the operation process (during tribological tests) are shown along with the defined tribological characteristics. The results include a surface map with the selected peaks/pits, surface axonometrics, and inversion of the surface axonometrics.

**Figure 3:**
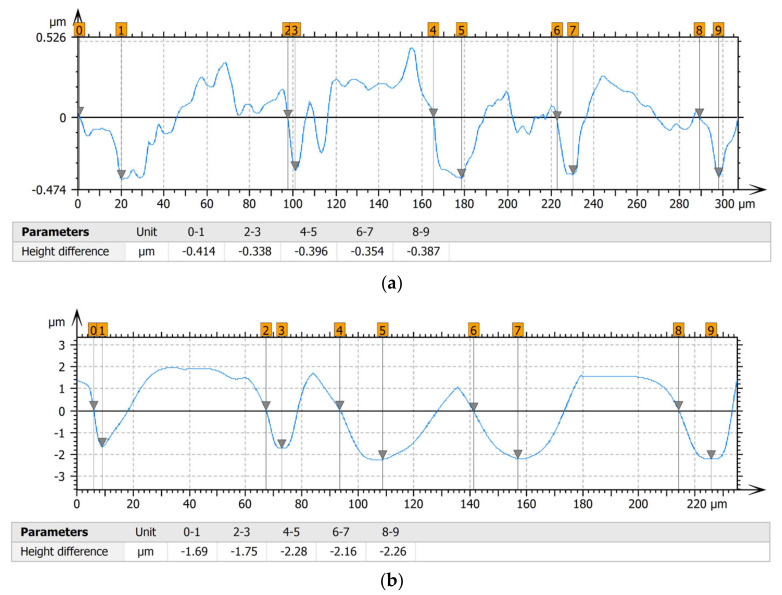
Valley characteristics: (**a**) Surface A, (**b**) Surface B.

**Figure 4:**
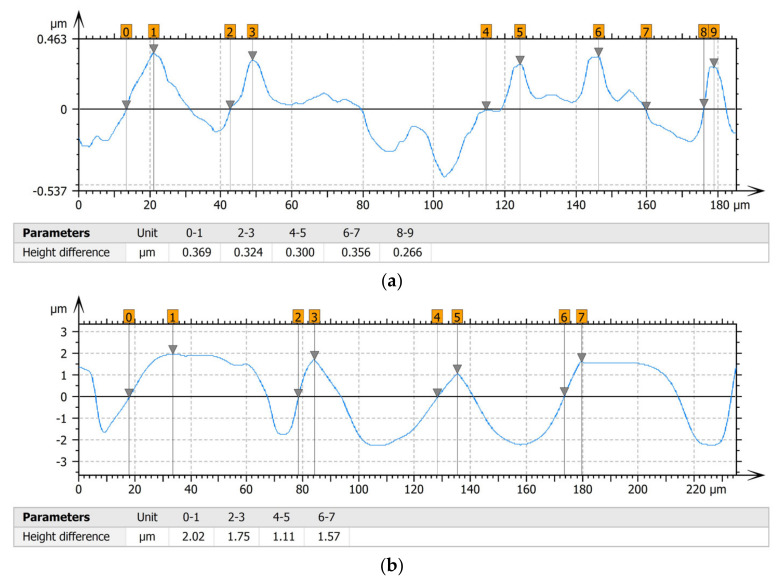
Peaks characteristics: (**a**) Surface A, (**b**) Surface B.

The analysis of the valleys (Figure 3) showed that on Surface A, their depth ranged from 0.338 µm to 0.414 µm, and on Surface B, it ranged from 1.69 µm to 2.28 µm. Additionally, some peaks (Figure 4) were observed on Surface A that ranged in height from 0.266 µm to 0.369 µm, and a lot of plateau peaks were observed on Surface B, which ranged in height from 1.11 µm to 2.02 µm.

Table 5 presents representative graphs of the Abbott–Firestone curve and Sk parameters: Sk—core roughness height, Spk—reduced summit height, Svk—reduced valley depth, Srm_1_—upper bearing area (peaks), Smr_2_—lower bearing area (pits). Additionally, in the same table, selected parameters describing the surface roughness of the studied samples are presented (ISO 25178): Sq—root mean square height, Ssk—skewness, Sku—kurtosis, Sp—maximum peak height, and Sv—maximum pit depth.

The Abbott–Firestone curve showed that in the case of Surface A, it was inclined at a significant angle compared to Surface B, which indicated a more even (stable) nature of the Surface B irregularities. The analysis of the Sk parameters showed that the value of Sk was only twice as large for Surface B. Much larger differences were noted for the parameters Spk and Svk. The value of the Spk was twelve times higher for Surface B, which indicated the presence of many high peaks on this surface (their share for Surfaces A and B were, respectively 8.24 and 15.6%), which could be removed by the surface of the cooperating element during the operation process. The value of the Svk was over four times higher than it was for Surface B, which indicated that there were valleys on this surface, the share of which one Surfaces A and B was 12.3 and 10.5%, respectively.

The value of the Sq parameter indicated that Surface B had greater roughness in comparison with surface A. The value of the Ssk parameter for Surface A was negative, which indicated that the surface had rather plateau-like characteristics as opposed to Surface B, for which the value of this parameter was positive. It can be assumed that Surface B was characterized by peaks with steep slopes and vertices with a small rounding radius. Taking into account the analysis of the Sk parameters, the shapes of the irregularities may play a key role in the operation process during tribological studies. The Sku parameter was almost three times larger for Surface B. High values of the Sku parameter testified to the occurrence chaotic features on the surface, such as pits or peaks, which is repeated in qualitative analysis. Parameters such as Sp and Sv reflected the nature of the irregularities on the studied Surfaces A and B, confirming the previous analysis.

**Figure 5:**
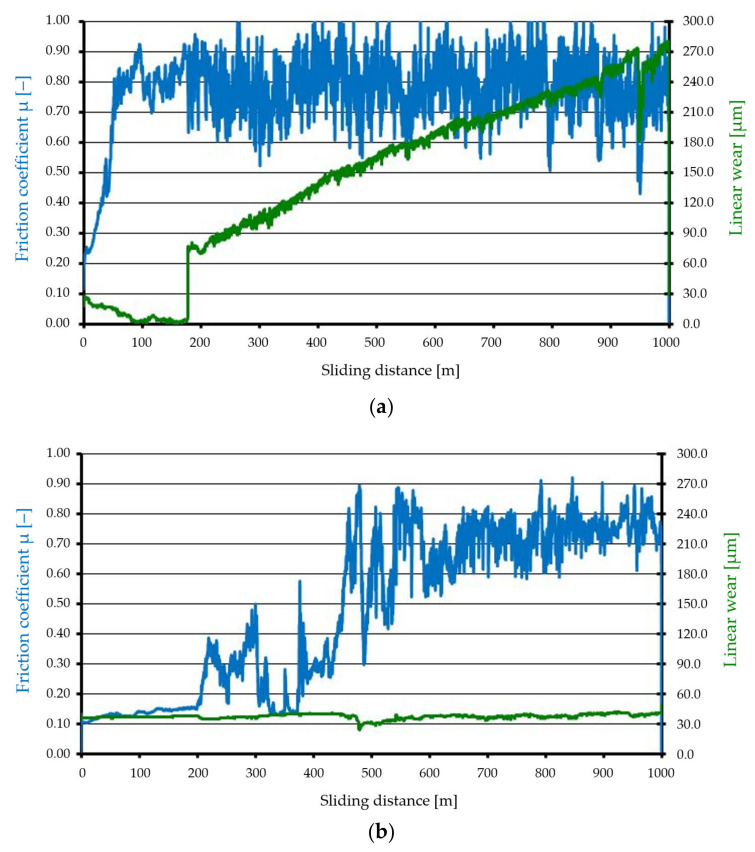
Tribological characteristics (studies without lubricant): (**a**) Surface A, (**b**) Surface B.

**Figure 6:**
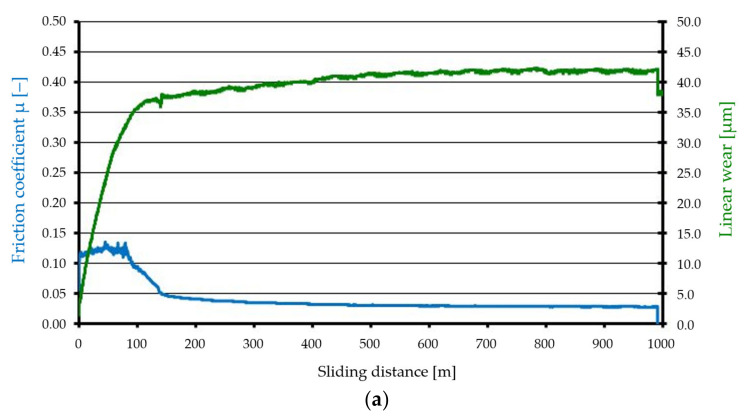

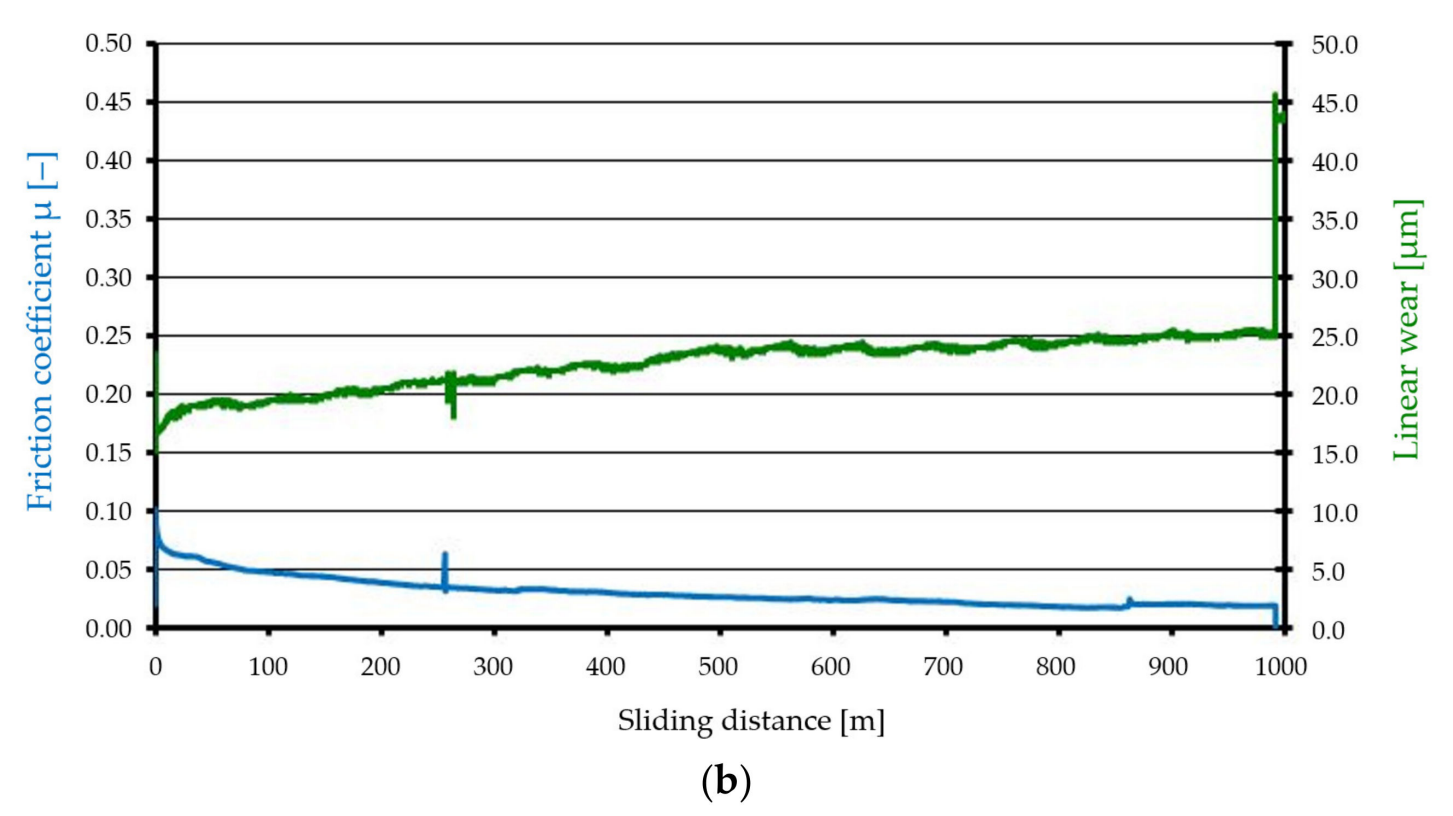
Tribological characteristics (studies with lubricant): (**a**) Surface A, (**b**) Surface B.

The results of tribological tests are presented in Figure 5 and Figure 6 as well as in Table 6. The figures show the variation of the friction coefficient *μ* and the linear wear as a function of the sliding distance *d.* Figure 5 shows the results of tests without lubrication, and Figure 6 shows the results of tests with lubrication.

Surface A, which was characterized by less surface roughness and that could be described by various parameters, showed a much more chaotic course of variation for the friction coefficient, which, after a run-in period (about 100 m), began to stabilise (remained at the same level, within a constant range of changes). At the beginning, the linear wear was stable (to approx. 180 m), followed by an increase until the end of the test (change in the range of approx. from 75 to 285 µm). Surface B, which was characterized by greater surface roughness, showed unstable changes in the friction coefficient (after 200 m of the sliding distance), while the linear wear from the beginning to the end of the test was stable and remained at a level of about 40 µm.

During the tribological tests with lubrication, Surface A initially showed (at the stage of the run-in period—the first 80 m) a friction coefficient with a stable course (about 0.13) followed by a sharp drop and stabilisation at the level of 0.03. At the beginning, the linear wear increased rapidly, and after about 120 m, it began to stabilize—the changes were small and ranged from about 37 to 42 µm. During the tribological tests with lubrication, Surface B showed a gradual decrease in the friction coefficient, which varied from about 0.1 to 0.02, while linear wear gradually increased from about 17 to 25 µm (in the end of test cycle). 

Table 6 presents the average values of the tribological characteristics, i.e., the friction coefficient *µ* and wear intensity *I*, together with the standard deviation.

The results presented in Table 6 show slight differences in the average friction coefficient (at a level equal to 0.0125) for the tribological tests without lubrication. Much larger differences were noted for the tribological tests with lubrication—the friction coefficient for Surface B was almost three times lower than it was for Surface A. In the case of the tribological tests with lubrication, the wear intensity was significantly lower compared to the tribological tests without lubrication, while this intensity for Surface A was higher.

## 4. Conclusions

Aluminum Matrix Composites reinforced with 10 wt.% of Al_2_SiO_5_ particles were manufactured by vacuum-assisted stir casting. As a result of a finishing process, the two different types of surface topography were obtained—Surfaces A and B.

The surface topography of the manufactured samples was studied using WLIM. Sur-face A was characterized by lower values for the 3D roughness parameters compared to Surface B. The differences were reflected in the results of the tribological tests, both those without lubricant and with lubricant.

The average friction coefficient for the tribological tests without lubrication for both A and B surfaces was comparable—the difference was small (at a level equal to 0.0125, smaller for Surface B). Much larger differences were noted for the tribological tests with lubrication—the friction coefficient for Surface B was almost three times lower than it was for Surface A. 

Surface A of the block characterized by less surface roughness displayed a greater density of summits (results of studies for manufactured surfaces) and the same increased contact with the co-acting surface of the ring. It is likely that the peaks on Surface A, which were densely spaced and had a regular shape, affected the greater bearing surface and consequently the greater friction coefficient as well as the intensity of wear of the ring despite their smaller size. 

In the case of the tribological tests with lubrication, the friction coefficient as well as the wear intensity was significantly lower compared to tribological tests without lubrication. The use of a PAO8 lubricant resulted in a significant reduction in the tribological characteristics for both Surfaces A and B. However, higher values of the tribological characteristics were still recorded for Surface A. The explanation for that phenomena may be the much larger pits (surface defects—valleys/cavities) on Surface B than on Surface A. These pits were where the lubricating fluid accumulated, which ensured the constant lubrication of the contact zone, providing better lubrication for Surface B and limited lubrication for Surface A. Similar results were obtained for another material—a titanium alloy used in medicine [39,40].

The results of the conducted studies proved that it is important to pay attention not only to the Ra parameter (2D parameter), which is often given in standards, but to other surface characteristics as well, including surface roughness parameters (3D parameters), the Abbott–Firestone curve, and its parameters, which could play an important role when interpreting the results of tribological studies. Studies that consider manufactured surface topographies are often disregarded or are only briefly analyzed, with researchers only be concerned with determining the Ra parameter or showing the surface morphology (images obtained using SEM or OM). It is important to check to what extent specific treatments will have a positive effect on the functional properties of the material and the parts that are made of it. Consequently, this affects the production cost, which is often unjustifiably high compared to the effects in use.

In order for the studied composite Al-10% Al_2_SiO_5_ to be successfully used in engineering solutions, in the future, the results should be verified by conducting tests on prototype elements in conditions reflecting actual working conditions.

## Figures and Tables

**Table 1:** Properties of the studied material.

Sample	Density (g/cm^3^)	Microhardness (HV)	Tensile Strength (MPa)	Impact Strength (J)
Pure Al	2.6871	40.53	70.67	9.70
Al-10%Al_2_SiO_5_	2.6763	41.87	89.20	11.75

**Table 2:** Characteristic of tribological tests.

Tribotester [36]	Sliding Joint	Parameters
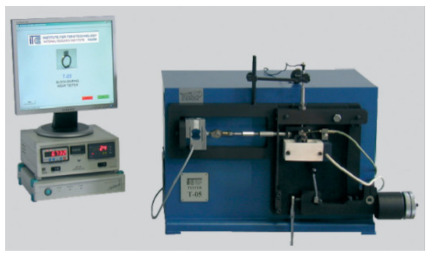	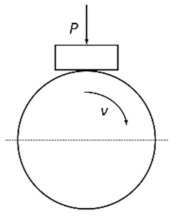	Contact geometry: non-conformal (line)
		Type of movement: sliding
		Friction pair (materials): composite ASRC (block)—steel 100Cr6 (ring)
		Normal load: *P* = 20 N
		Sliding velocity: *v* = 0.1 m/s
		Sliding distance (test cycle): *d* = 1000 m
		Lubrication: without or with lubricant PAO8 (polyalphaolefin, synthetic oil)

**Table 3:** Results of manufactured surface topography.

	**Surface A**	**Surface B**
Photo	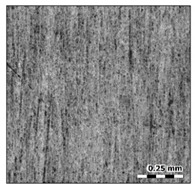	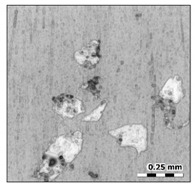
Surface map	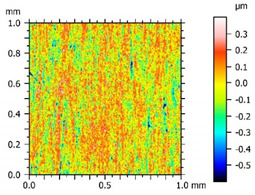	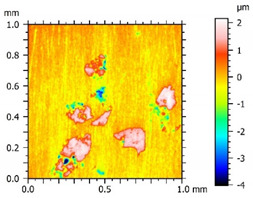
Surface axonometric	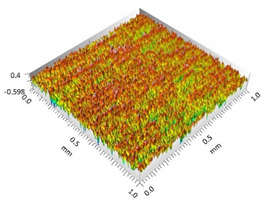	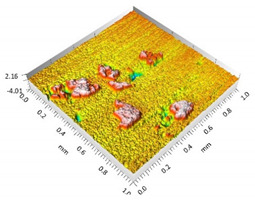

**Table 4:** Results of manufactured surface topography—characteristic peaks or pits.

	Surface A	Surface B
Surface map	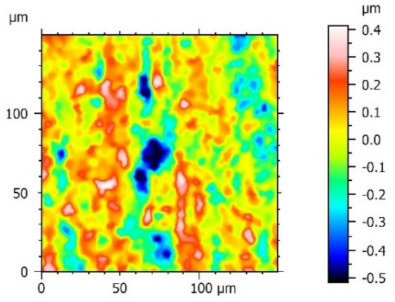	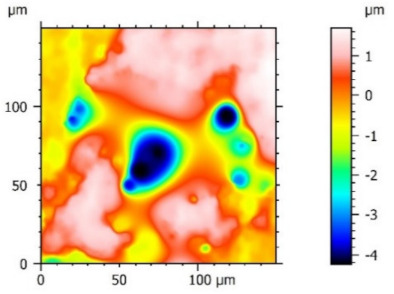
Surface axonometric	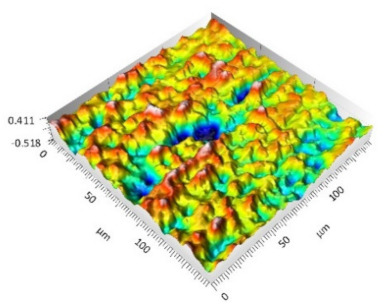	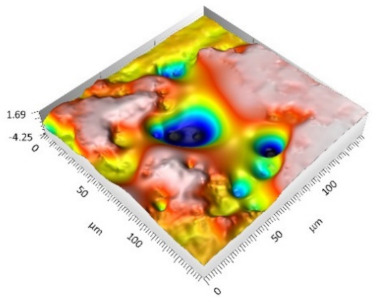
Inversion of surface axonometric	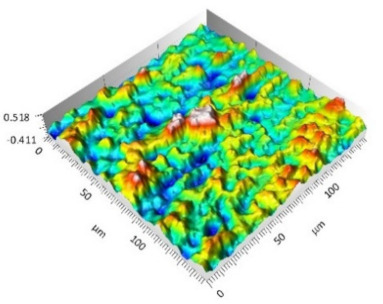	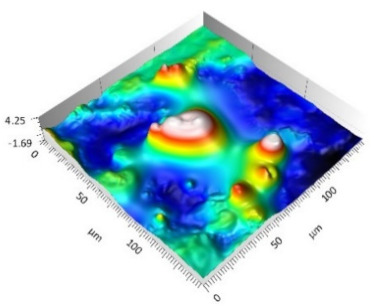

**Table 5:** Results of manufactured surface topography—Abbott–Firestone curve and parameters.

Surface A					Surface B				
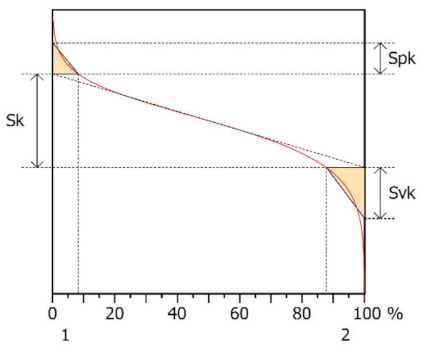					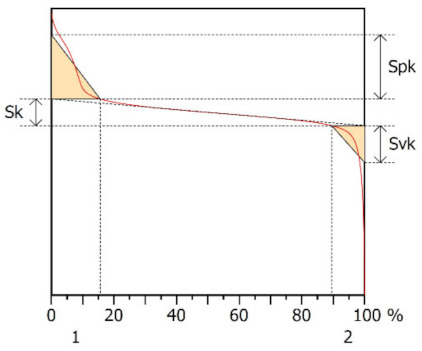				
Abbott–Firestone curve—Sk parameters									
Sk [µm]	Spk [µm]	Svk [µm]	Smr1 [%]	Smr2 [%]	Sk [µm]	Spk [µm]	Svk [µm]	Smr1 [%]	Smr2 [%]
0.329	0.109	0.180	8.24	87.7	0.574	1.38	0.796	15.6	89.5
Surface texture—3D parameters									
Sq [µm]	Ssk [-]	Sku [-]	Sp [µm]	Sv [µm]	Sq [µm]	Ssk [-]	Sku [-]	Sp [µm]	Sv [µm]
0.136	−0.462	3.69	0.400	0.598	0.542	0.527	9.89	2.16	4.01

**Table 6:** Tribological characteristics—average value with standard deviation.

Lubrication	Surface A	Surface B
–	*µ* = 0.7632 ± 0.0416	*µ* = 0.7507 ± 0.0429
	*I* = 0.2498 ± 0.0210 [µm/cycle]	*I* = 0.21033 ± 0.0168 [µm/cycle]
PAO8	*µ* = 0.0802 ± 0.0122	*µ* = 0.0294 ± 0.0165
	*I* = 0.0099 ± 0.0018 [µm/cycle]	*I* = 0.0075 ± 0.0041 [µm/cycle]

## Data Availability

Data is contained within the article.
